# Supplementary material for: Activation of Persulfate for Degrading Tetracycline Using the Leaching Residues of the Lead-Zinc Flotation Tailing
Source: Polymers (Basel). 2022 Jul 21;14(14):2959. doi: 10.3390/polym14142959 (PMC9316694; doi:10.3390/polym14142959)
Supplement: Supplementary file 1 [file polymers-14-02959-s001.zip › polymers-1809703-Supplementary material.pdf]

*Supporting Information*

# Activation of Persulfate for Degrading Tetracycline using the Leaching Residues of the Lead-zinc Flotation Tailing

Jun Wang[1], XiaoCui Wen[2,3], ShaoJun Jiang[2,3], Tao Chen[2,3,*]

[1] Fankou Lead-Zinc Mine,Shenzhen Zhongjin Lingnan Non-ferrous metal Company Limited, ShaoGuan, 512000, China; 9874136@qq.com( J.Wang)
[2] School of Environment, South China Normal University, University Town, Guangzhou 510006, China ; xiaocui.wen@m.scnu.edu.cn (X.C. Wen), shaojunj93@163.com (S.J. Jiang), tao.chen@m.scnu.edu.cn (T.Chen)
[3] Guangdong Provincial Key Laboratory of Chemical Pollution and Environmental Safety & MOE Key Laboratory of Theoretical Chemistry of Environment, South China Normal University, Guangzhou 510006, China

**Text S1** Effects of operational parameters on TC degradation

According to different influencing factors of the study, such as PS concentration, pH value, TSM dosage and TC concentration, four kinds of experiments on the degradation of TC were set up (Xu et al.,2019; Zhu et al.,2019). The first experiment was studying the effect of PS concentration. When other conditions were essentially the same, one reaction systems by using TSM as catalysts were set up. The experiment was carried out in test cups in which the concentrations of PS were 0, 2.5, 5, 10 and 20 mM. The second experiment to study the effect of pH value was adjusting the solution pH in multiple test cups with the same amounts of PS (i.e., 5 mM) to make the pH fall in range of 4-12 by 0.1 M HCl or NaOH aqueous solutions (which must contain pH= 7). The effect of the catalyst dosage on tetracycline degradation was studied. The amount of catalyst was 0, 0.05, 0.1, 0.2 and 0.4 g/L respectively, and it was carried out in the environment of pH=7.0 and PS of 5 mM. Finally, the effect of the initial TC concentration on TC degradation was also studied. The concentrations of PS were 5, 10, 20, 30 and 50 mg/L and the other conditions were essentially the same. Except for tests of pH effect, all experiments were performed without adjusting pH value, in order to avoid the influences of buffer salt or

other ionic. Each experiment was run in triplicates.

References

Zhu. K, Xu. H, Chen. C, Ren. X, Alsaedi. A, Hayat. T.(2019) Encapsulation of Fe0-dominated Fe3O4/Fe0/Fe3C nanoparticles into carbonized polydopamine nanospheres for catalytic degradation of tetracycline via persulfate activation, Chem. Eng. J. 372: 304–311.

Xu. X, Yang. Y, Jia. Y, Lian. X, Zhang. Y, Feng. F, Liu. Q, Xi. B, Jiang. Y. (2019). Heterogeneous catalytic degradation of 2,4-dinitrotoluene by the combined persulfate and hydrogen peroxide activated by the as-synthesized Fe-Mn binary oxides, Chem. Eng. J. 374 776–786

Tables and Figures captions

Table S1 Chemical composition and heavy metals content of the leaching residue

| Component | $SiO_2$ | $Al_2O_3$ | $Fe_2O_3$ | CaO | MgO | $Na_2O$ | $K_2O$ |
|---|---|---|---|---|---|---|---|
| Content (%) | 81.54 | 3.16 | 5.94 | 0.98 | 0.44 | 0.94 | 0.85 |
| Component | Pb | Cu | Cd | Cr | Hg | As | Available $SiO_2$ |
| Content (%) | 0.0165 | 0.0046 | 0.0001 | 0.0175 | 0.0001 | 0.0050 | 0.88 |

Table S2 Water quality index data

| | Milli-Q water | Tap-water | River water |
|---|---|---|---|
| pH | 6.84 | 7.54 | 7.04 |
| EC μS/cm | 287.61 | 43.59 | 1.03 |
| $Mn^{2+}$(mg/L) | 0.053 | - | - |
| COD (mg/L) | 65.47 | - | - |

| | | | |
|---|---|---|---|
| $NH_4^+$-N （mg/L） | 1.25 | - | - |

EC, mean Electric Conductivity；“-”mean Not detected.

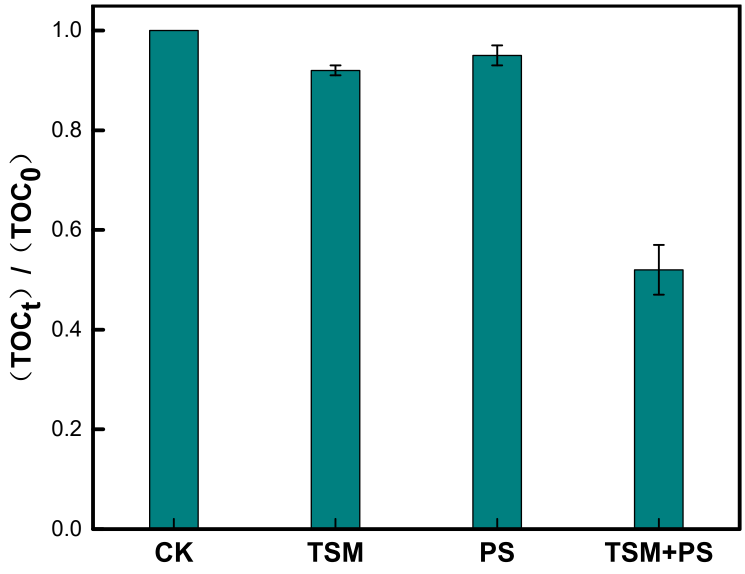


Figure S1 Change in the TOC during TC degradation

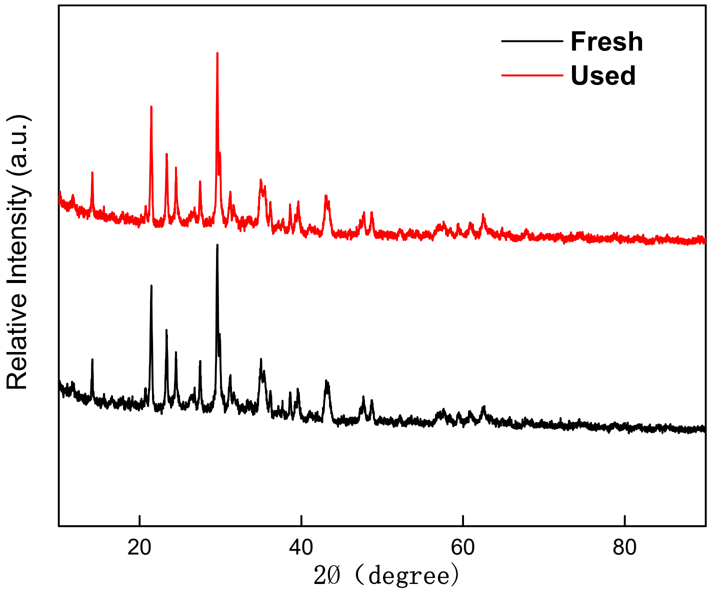


Figure S2. XRD patterns of the TSM samples before and after reaction system

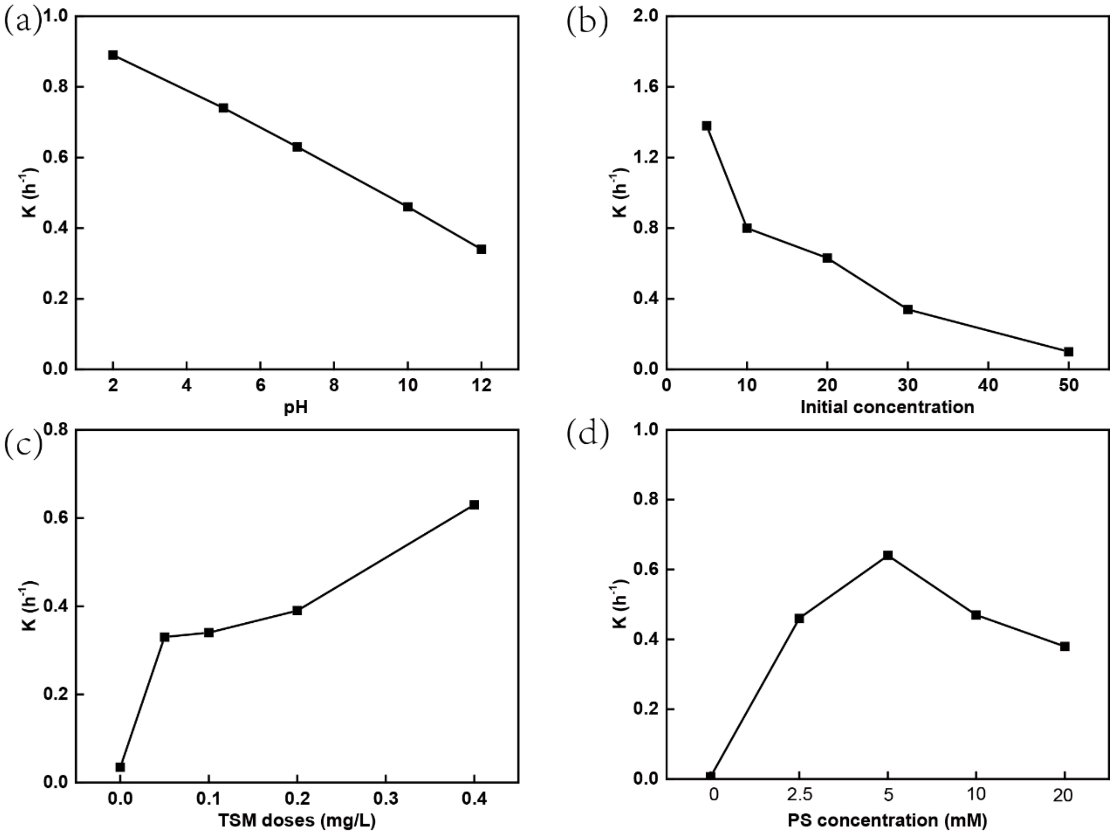


Figure S3 Effects of different parameter on TC degradation. ( (a) the pseudo first-order rate constant of pH; (b) the pseudo first-order rate constant of initial concentration of TC; (c) The pseudo first-order rate constant of TSM dosage and (d) the pseudo first-order rate constant of PS concentration). (Reaction conditions: (a) PS = 5 mM, pH 7, TC = 20 mg/L; (b) PS = 5 mM, TSM=0.4 g/L, TC = 20 mg/L; (c) TC=20mg/L, pH=7, TSM=0.4 g/L;(d) TC=20 mg/L, pH=7, TSM=0.4 g/L)

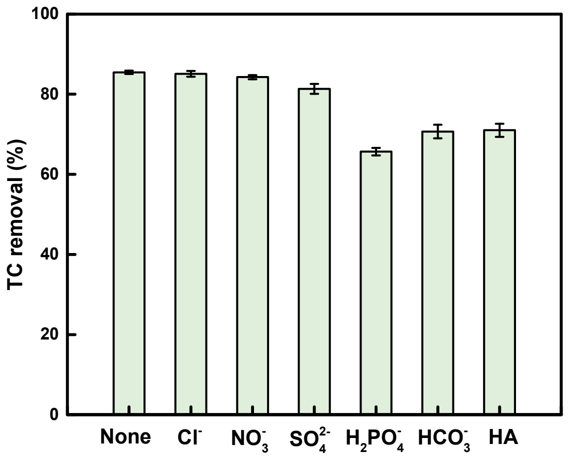


Figure S4 Effect of common components in natural waters on TC removal by the TSM/PS system. (Reaction conditions: TC 20 mg/L, PS 5 mM, TSM 0.4 g/L, and initial pH=7)

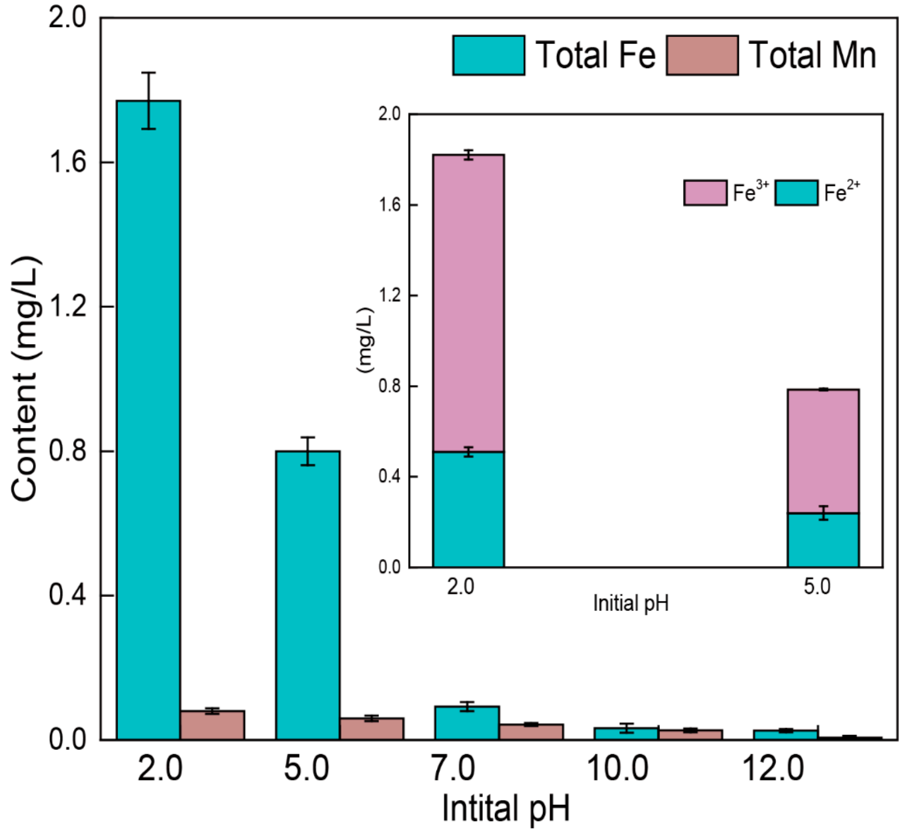


Figure S5. Leaching of Fe and Mn in TSM/PS system at different pH; and Different iron content ($Fe^{2+}$ and $Fe^{3+}$) under at pH conditions (illustration)
